# Assessment of Second Primary Cancer Risk Among Men Receiving Primary Radiotherapy vs Surgery for the Treatment of Prostate Cancer

**DOI:** 10.1001/jamanetworkopen.2022.23025

**Published:** 2022-07-28

**Authors:** Hilary P. Bagshaw, Katherine D. Arnow, Amber W. Trickey, John T. Leppert, Sherry M. Wren, Arden M. Morris

**Affiliations:** 1 Department of Radiation Oncology, Stanford University, Palo Alto, California; 2 Department of Surgery, Stanford University, Palo Alto, California; 3 Stanford–Surgery Policy Improvement Research and Education Center, Stanford University, Palo Alto, California; 4 Department of Urology, Stanford University, Palo Alto, California; 5 Palo Alto Veterans Health Care System, Palo Alto, California

## Abstract

**Importance:**

Shared decision-making is an important part of the treatment selection process among patients with prostate cancer. Updated information is needed regarding the long-term incidence and risk of second primary cancer after radiotherapy vs nonradiotherapy treatments, which may help to inform discussions of risks and benefits for men diagnosed with prostate cancer.

**Objective:**

To assess the current incidence and risk of developing a second primary cancer after receipt of radiotherapy vs nonradiotherapy treatments for prostate cancer.

**Design, Setting, and Participants:**

This retrospective cohort study used the Veterans Affairs Corporate Data Warehouse to identify 154 514 male veterans 18 years and older who had localized prostate cancer (tumor stages T1-T3) diagnosed between January 1, 2000, and December 31, 2015, and no cancer history. A total of 10 628 patients were excluded because of (1) incomplete treatment information for the year after diagnosis, (2) receipt of both radiotherapy and a surgical procedure in the year after diagnosis, (3) receipt of radiotherapy more than 1 year after diagnosis, (4) occurrence of second primary cancer or death within 1 year or less after diagnosis, (5) prostate-specific antigen value greater than 99 ng/mL within 6 months before diagnosis, or (6) no recorded Veterans Health Administration service after diagnosis. The remaining 143 886 patients included in the study had a median (IQR) follow-up of 9 (6-13) years. Data were analyzed from May 1, 2021, to May 22, 2022.

**Main Outcomes and Measures:**

Diagnosis of a second primary cancer more than 1 year after prostate cancer diagnosis.

**Results:**

Among 143 886 male veterans (median [IQR] age, 65 [60-71] years) with localized prostate cancer, 750 (0.5%) were American Indian or Alaska Native, 389 (0.3%) were Asian, 37 796 (26.3%) were Black or African American, 933 (0.6%) were Native Hawaiian or other Pacific Islander, 91 091 (63.3%) were White, and 12 927 (9.0%) were of unknown race; 7299 patients (5.1%) were Hispanic or Latino, 128 796 (89.5%) were not Hispanic or Latino, and 7791 (5.4%) were of unknown ethnicity. A total of 52 886 patients (36.8%) received primary radiotherapy, and 91 000 (63.2%) did not. A second primary cancer more than 1 year after prostate cancer diagnosis was present in 4257 patients (3.0%), comprising 1955 patients (3.7%) in the radiotherapy cohort and 2302 patients (2.5%) in the nonradiotherapy cohort. In the multivariable analyses, patients in the radiotherapy cohort had a higher risk of second primary cancer compared with those in the nonradiotherapy cohort at years 1 to 5 after diagnosis (hazard ratio [HR], 1.24; 95% CI, 1.13-1.37; *P* < .001), with higher adjusted HRs in the subsequent 15 years (years 5-10: 1.50 [95% CI, 1.36-1.65; *P* < .001]; years 10-15: 1.59 [95% CI, 1.37-1.84; *P* < .001]; years 15-20: 1.47 [95% CI, 1.08-2.01; *P* = .02).

**Conclusions and Relevance:**

In this cohort study, patients with prostate cancer who received radiotherapy were more likely to develop a second primary cancer than patients who did not receive radiotherapy, with increased risk over time. Although the incidence and risk of developing a second primary cancer were low, it is important to discuss the risk with patients during shared decision-making about prostate cancer treatment options.

## Key Points

**Question:**

Do patients with prostate cancer in the modern era have a higher incidence and risk of developing a second primary cancer after radiotherapy compared with nonradiotherapy treatments?

**Findings:**

In this cohort study of 143 886 patients with localized prostate cancer, receipt of radiotherapy was associated with a small but statistically significant increase in the risk of developing a second primary cancer.

**Meaning:**

This study’s findings suggest that it is important that shared decision-making among patients with prostate cancer includes discussion of late toxic effects, including the risk of developing a second primary cancer, and that the possibility of developing a second primary cancer after radiotherapy be considered during evaluation of symptoms among survivors.

## Teaser

This cohort study uses data from the Veterans Affairs Corporate Data Warehouse to assess the incidence and risk of developing a second primary cancer after receipt of radiotherapy vs nonradiotherapy treatments among men with prostate cancer.

## Introduction

Localized prostate cancer is a distinctly preference-sensitive condition because radical prostatectomy, radiotherapy, and, in some cases, active surveillance have equivalent long-term cancer-specific outcomes.^1^ The risk profiles of each treatment option differ; therefore, shared decision-making is an important part of the prostate cancer treatment approach.^1^ Patients need to feel confident in understanding how the risks and benefits pertain to them. Given that all treatment options are associated with excellent prostate cancer–specific mortality rates,^2^ discussion of the potential adverse effects of surgical procedures and radiotherapy is especially important to help patients select treatment that best suits their goals.

The risks associated with radical prostatectomy are well understood because of the availability of many surgical outcomes data sets that cover the brief perioperative period, during which complications become apparent. During radiotherapy, clinicians similarly assess patients for the presence of acute toxic effects and discuss symptom management. However, late toxic effects can present months to years after completion of radiotherapy when patients may no longer regularly receive care from a radiation oncologist.

One late toxic effect, radiotherapy-induced cancer, specifically sarcoma occurring in previously irradiated bone, was first described in the 1940s.^3^ The original definition of radiotherapy-induced cancer, still in use today, is a cancer that (1) occurs in an irradiated field, (2) has a latency period of at least 4 years between completion of radiotherapy and diagnosis of the new cancer, (3) has different histological features than the primary cancer, and (4) was not present before radiotherapy exposure.^3,4^ If all required elements of this definition cannot be confirmed, the term *second primary cancer* is used to describe cancer diagnosed in patients who previously received radiotherapy. In this study, we were not able to unequivocally confirm part 4 of the definition of a radiotherapy-induced cancer; therefore, we referred to a cancer diagnosed in a previously irradiated field as a second primary cancer.

Prostate radiotherapy exposes the bladder, rectum, and other nearby structures, including bone marrow, to high doses of radiation; however, data regarding second primary cancers in these organs are conflicting. An increased risk of bladder cancer has been reported among patients with prostate cancer who received radiotherapy compared with those who did not,^5^ which was similar to risks reported in the cervical and ovarian cancer literature.^6,7,8^ An older study found an increased risk of second primary rectal cancers that was higher than the background rate,^9^ whereas more current data^10,11^ have suggested this risk is no higher than that found in the general population. Hematologic cancers historically have not been associated with prostate radiotherapy^5^; however, they have been associated with pelvic radiotherapy for the treatment of other cancers.^6^

To update the assessment of long-term risk of developing a second primary cancer after receipt of primary radiotherapy for the treatment of localized prostate cancer, we examined patients diagnosed with prostate cancer from January 1, 2000, to December 31, 2015, who were receiving care from the Veterans Affairs (VA) health care system. Data were analyzed from May 1, 2021, to May 22, 2022. We hypothesized that the incidence and risk of developing a second primary cancer within the relevant anatomical field would be higher among patients who received primary radiotherapy compared with those who received nonradiotherapy treatments, including surgical procedures, active surveillance, medical management, or observation.

## Methods

### Data Source

We conducted a retrospective analysis of the incidence and risk of developing a second primary cancer after treatment for prostate cancer using data from the VA Corporate Data Warehouse (CDW). The Stanford Institutional Review Board approved this study. A waiver of informed consent was granted because the retrospective study design posed minimal risk to participants. This study followed the Strengthening the Reporting of Observational Studies in Epidemiology (STROBE) reporting guideline for cohort studies.

We identified patients and captured clinical and treatment characteristics in the CDW oncology data set, which includes cancer diagnoses, treatment information, and clinical characteristics of patients nationally from VA medical centers that diagnose or treat cancer. We supplemented clinical information from the CDW oncology data set with patient Veterans Health Administration electronic health record information using the Observational Medical Outcomes Partnership Common Data Model,^12^ which provides standardized terms for clinical data to facilitate research across multiple systems and periods.

### Study Cohort

We identified men 18 years and older with incident localized prostate cancer (tumor stages T1-T3) diagnosed between 2000 and 2015 and no cancer history in the CDW oncology data set. We excluded patients who (1) had incomplete treatment information for the year after diagnosis, (2) received both radiotherapy and a surgical procedure in the year after diagnosis (excluding adjuvant or salvage radiotherapy), (3) started radiotherapy more than 1 year after diagnosis, (4) developed a second primary cancer of interest or died within 1 year after diagnosis, (5) had a recorded prostate-specific antigen (PSA) value greater than 99 ng/mL in the 6 months before diagnosis (to convert from nanograms per milliliter to micrograms per liter, multiply by 1), or (6) did not have a recorded Veterans Health Administration service date (initiation date of health care encounter) after diagnosis. We compared patients who received primary radiotherapy in the year after diagnosis (radiotherapy cohort) with their counterparts who received a primary surgical procedure, active surveillance, medical management, or observation (nonradiotherapy cohort).

### Outcomes

Our primary outcome was development of a second primary cancer. Relevant cancer types were determined by expert review (H.P.B., J.T.L., S.M.W., and A.M.M.) of the cancer site documented in the CDW oncology file of the identified cohort and included cancers of organs potentially in the radiotherapy field, including bladder cancer, rectal cancer, pelvic soft tissue cancer, cancer of the male genitalia (excluding the testes), bone cancer, cancer of the hematoreticular system, leukemia, and lymphoma. A total of 3966 second primary cancers (93.2%) were verified by laboratory and/or histopathological analysis.

### Covariates

Patient demographic and clinical covariates were determined a priori based on clinical importance. Demographic covariates of interest included age at diagnosis, race, ethnicity, and median income and educational level based on patient zip code at the time of diagnosis. Clinical characteristics included diagnosis year; Agent Orange exposure (yes or no); D’Amico risk classification (low, intermediate, or high likelihood of prostate cancer recurrence),^13^ which includes Gleason score (1-10, with higher scores indicating greater likelihood of cancer growth and spread), PSA value (with higher values indicating greater likelihood of prostate cancer), and clinical tumor stage (T1-T4, with T1 indicating the tumor cannot be felt during a direct rectal examination or observed on imaging but may be detected when a surgical procedure is performed for another condition, T2 indicating the tumor appears to be confined to the prostate, T3 indicating the tumor has grown outside the prostate and may have spread to the seminal vesicles, and T4 indicating the tumor has spread to tissues next to the prostate other than the seminal vesicles); and score on the Prostate Cancer Comorbidity Index^14^ (a weighted scale based on age and comorbidity that was validated among men with prostate cancer to estimate long-term noncancer mortality; scores were categorized as 0, 1-2, 3-4, and ≥5, with higher scores indicating older age and greater number of comorbidities), which was calculated using electronic health record data. Missing data on PSA values or Agent Orange exposure in the CDW oncology data set were supplemented by searching the health record for the most recent PSA value in the 6 months before diagnosis or any indication of Agent Orange exposure. A *missing* category was added for variables with incomplete data. A substantial amount of data on smoking status at diagnosis was missing in early diagnosis years (eg, 66% missing in 2000); smoking status was therefore incorporated only in a sensitivity analysis. We categorized smoking status into never, former, and current based on the most frequently occurring response before diagnosis, as previously described.^15^

### Statistical Analysis

We compared demographic and clinical characteristics between patients in the radiotherapy and nonradiotherapy treatment cohorts using χ^2^ tests for categorical variables and Wilcoxon rank sum tests for continuous variables. We assessed unadjusted survival in each treatment group using a life table. We used Cox regression analysis to estimate the association between radiotherapy and the risk of a second primary cancer, adjusted for all covariates of interest. To test the proportional hazard assumption, we included an interaction term between treatment and time. The interaction was statistically significant, suggesting the treatment effect changed over time; therefore, we calculated a piecewise model with radiotherapy effect assessed at 5-year intervals. Time to event was defined as time from the diagnosis of prostate cancer to the diagnosis of a second primary cancer. Patients without a second primary cancer were censored at the date of their last VA visit or the date of death through December 31, 2020.

To compare men who were eligible for active therapy, we performed a sensitivity analysis comparing patients in the radiotherapy cohort with patients in the nonradiotherapy cohort who received a surgical procedure (excluding patients who received active surveillance, medical management, or observation because these patients may have had more medical comorbidities than those eligible for active treatment) using a piecewise Cox model adjusted for the same covariates included in the main analysis. We performed 3 additional sensitivity analyses to confirm no difference in the primary end point. The first sensitivity analysis included only patients with available smoking data (with smoking status as an additional covariate in the Cox model), the second included hormone therapy use as a covariate, and the third included only patients with PSA values lower than 40 ng/mL.

Statistical significance was set at 2-tailed *P* = .05. Analyses were performed using Stata MP software, version 15.1 (StataCorp LLC), and R software, version 4.1.2 (R Foundation for Statistical Computing).

## Results

**Figure 1.**
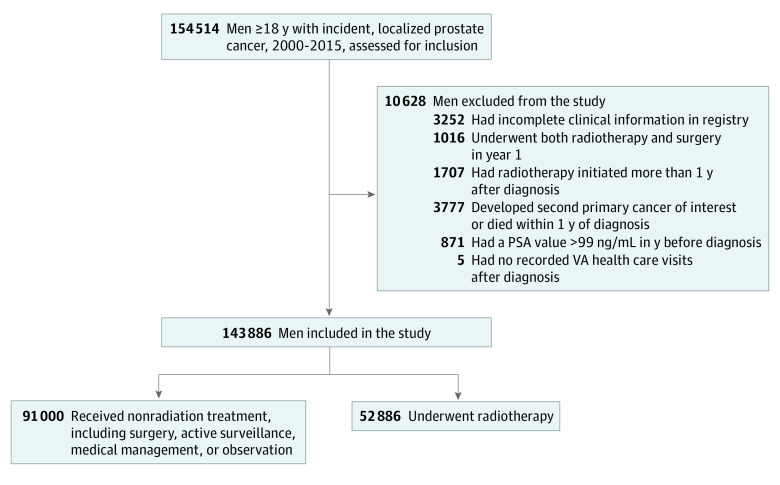
Study Inclusion and Exclusion Criteria To convert PSA from nanograms per milliliter to micrograms per liter, multiply by 1. PSA indicates prostate-specific antigen; and VA, Veterans Affairs.

Among 143 886 male veterans with localized prostate cancer who met inclusion criteria (Figure 1), the median (IQR) age was 65 (60-71) years. A total of 750 patients (0.5%) were American Indian or Alaska Native, 389 (0.3%) were Asian, 37 796 (26.3%) were Black or African American, 933 (0.6%) were Native Hawaiian or other Pacific Islander, 91 091 (63.3%) were White, and 12 927 (9.0%) were of unknown race; 7299 patients (5.1%) were Hispanic or Latino, 128 796 (89.5%) were not Hispanic or Latino, and 7791 (5.4%) were of unknown ethnicity. In the year after prostate cancer diagnosis, 52 886 patients (36.8%) received primary radiotherapy, and 91 000 (63.2%) did not receive primary radiotherapy. Among those who did not receive radiotherapy, 31 218 patients (34.3%) received a surgical procedure, and 59 782 patients (65.7%) received active surveillance, medical management, or observation. Demographic and clinical characteristics of the radiotherapy and nonradiotherapy cohorts at the time of prostate cancer diagnosis are shown in Table 1. The median (IQR) age was similar between the cohorts (66 [61-71] years in the radiotherapy cohort vs 65 [60-72] years in the nonradiotherapy cohort). Patients in the radiotherapy cohort vs the nonradiotherapy cohort had higher Gleason scores (eg, Gleason score >8 points: 8601 patients [16.3%] vs 8944 patients [9.8%]; *P* < .001) and higher clinical tumor stages (eg, stages T2 and T3: 17 089 patients [32.3%] vs 25 837 patients [28.4%]; *P* < .001). A greater proportion of patients in the radiotherapy cohort were Black or African American (14 754 patients [27.9%]) compared with the nonradiotherapy cohort (23 042 patients [25.3%]; *P* < .001).

**Table 1.** Participant Characteristics

Characteristic	Patients, No. (%)			*P* value
	Total (N = 143 886)	Nonradiotherapy cohort (n = 91 000)	Radiotherapy cohort (n = 52 886)	
Age at diagnosis, median (IQR), y	65 (60-71)	65 (60-72)	66 (61-71)	.14
Race				
American Indian or Alaska Native	750 (0.5)	486 (0.5)	264 (0.5)	<.001
Asian	389 (0.3)	281 (0.3)	108 (0.2)	
Black or African American	37 796 (26.3)	23 042 (25.3)	14 754 (27.9)	
Native Hawaiian or other Pacific Islander	933 (0.6)	594 (0.7)	339 (0.6)	
White	91 091 (63.3)	58 300 (64.1)	32 791 (62.0)	
Unknown	12 927 (9.0)	8297 (9.1)	4630 (8.8)	
Ethnicity				
Hispanic or Latino	7299 (5.1)	5276 (5.8)	2023 (3.8)	<.001
Not Hispanic or Latino	128 796 (89.5)	80 561 (88.5)	48 235 (91.2)	
Unknown	7791 (5.4)	5163 (5.7)	2628 (5.0)	
Median income based on patient zip code, $				
<39 000	44 969 (31.3)	28 215 (31.0)	16 754 (31.7)	.004
39 000-47 999	35 584 (24.7)	22 530 (24.8)	13 054 (24.7)	
48 000-63 999	35 891 (24.9)	22 644 (24.9)	13 247 (25.0)	
≥64 000	23 437 (16.3)	15 035 (16.5)	8402 (15.9)	
Missing	4005 (2.8)	2576 (2.8)	1429 (2.7)	
Median educational level based on patient zip code				
High school or less	71 780 (49.9)	44 715 (49.1)	27 065 (51.2)	<.001
Some college	59 554 (41.4)	38 062 (41.8)	21 492 (40.6)	
College or higher	8716 (6.1)	5756 (6.3)	2960 (5.6)	
Missing	3836 (2.7)	2467 (2.7)	1369 (2.6)	
PCCI score^a^				
0	59 881 (41.6)	38 901 (42.7)	20 980 (39.7)	<.001
1-2	21 369 (14.9)	13 366 (14.7)	8003 (15.1)	
3-4	14 688 (10.2)	9133 (10.0)	5555 (10.5)	
≥5	47 948 (33.3)	29 600 (32.5)	18 348 (34.7)	
D’Amico risk category^b^				
Low	50 454 (35.1)	35 234 (38.7)	15 220 (28.8)	<.001
Intermediate	52 751 (36.7)	31 413 (34.5)	21 338 (40.3)	
High	34 106 (23.7)	19 516 (21.4)	14 590 (27.6)	
Undefined	6575 (4.6)	4837 (5.3)	1738 (3.3)	
PSA, ng/mL				
<10	102 722 (71.4)	65 542 (72.0)	37 180 (70.3)	<.001
10-20	21 647 (15.0)	12 799 (14.1)	8848 (16.7)	
>20	12 611 (8.8)	8074 (8.9)	4537 (8.6)	
Missing	6906 (4.8)	4585 (5.0)	2321 (4.4)	
Gleason score^c^				
≤6	56 755 (39.4)	39 739 (43.7)	17 016 (32.2)	<.001
7	49 191 (34.2)	28 546 (31.4)	20 645 (39.0)	
≥8	17 545 (12.2)	8944 (9.8)	8601 (16.3)	
Missing	20 395 (14.2)	13 771 (15.1)	6624 (12.5)	
Clinical tumor stage^d^				
T1	97 232 (67.6)	61 976 (68.1)	35 256 (66.7)	<.001
T2	42 337 (29.4)	25 522 (28.0)	16 815 (31.8)	
T3	589 (0.4)	315 (0.3)	274 (0.5)	
Missing	3728 (2.6)	3187 (3.5)	541 (1.0)	

Abbreviations: PCCI, Prostate Cancer Comorbidity Index; PSA, prostate-specific antigen.

SI conversion factor: To convert PSA from nanograms per milliliter to micrograms per liter, multiply by 1.

^a^ Higher scores indicate older age and greater number of comorbidities.

^b^ Likelihood of prostate cancer recurrence based on Gleason score, PSA value, and clinical tumor stage.

^c^ Range, 1-10, with higher scores indicating greater likelihood of cancer growth and spread.

^d^ T1 indicates the tumor cannot be felt during a direct rectal examination or observed on imaging but may be detected when a surgical procedure is performed for another condition, T2 indicates the tumor appears to be confined to the prostate, and T3 indicates the tumor has grown outside the prostate and may have spread to the seminal vesicles but has not spread to other tissues next to the prostate.

**Figure 2.**
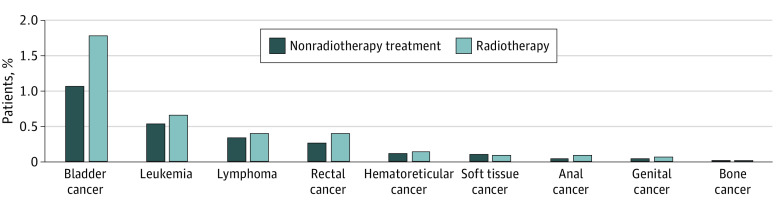
Most Frequent Second Primary Cancer Sites Among Men With Prostate Cancer

Over a median (IQR) follow-up of 9 (6-13) years, 4257 patients (3.0%) were diagnosed with a second primary cancer more than 1 year after their prostate cancer diagnosis, comprising 1955 patients (3.7%) in the radiotherapy cohort and 2302 patients (2.5%) in the nonradiotherapy cohort. The most frequent types of second primary cancer were bladder cancer (radiotherapy cohort: 957 patients [1.8%]; nonradiotherapy cohort: 985 patients [1.1%]), leukemia (radiotherapy cohort: 355 patients [0.7%]; nonradiotherapy cohort: 489 patients [0.5%]), lymphoma (radiotherapy cohort: 214 patients [0.4%]; nonradiotherapy cohort: 307 patients [0.3%]), rectal cancer (radiotherapy cohort: 219 patients [0.4%]; nonradiotherapy cohort: 244 patients [0.3%]), hematoreticular cancer (radiotherapy cohort: 76 patients [0.1%]; nonradiotherapy cohort: 98 patients [0.1%]), soft tissue cancer (radiotherapy cohort: 49 patients [0.1%]; nonradiotherapy cohort: 93 patients [0.1%]), anal cancer (radiotherapy cohort: 45 patients [0.1%]; nonradiotherapy cohort: 40 patients [0.04%]), male genital cancer (radiotherapy cohort: 30 patients [0.1%]; nonradiotherapy cohort: 33 patients [0.04%]), and bone cancer (radiotherapy cohort: 10 patients [0.02%]; nonradiotherapy cohort: 13 patients [0.01%]) (Figure 2).

The median (IQR) time to development of any second primary cancer was 6 (3-9) years after the prostate cancer diagnosis (median [IQR], 6.2 [3.5-9.5] years in the radiotherapy cohort and 5.5 [3.2-8.7] years in the nonradiotherapy cohort). The median (IQR) time to development of the 4 most frequent second primary cancers in the radiotherapy cohort was 6.2 (3.5-9.4) years for bladder cancer, 5.8 (3.6-9.2) years for leukemia, 5.7 (3.6-8.7) years for lymphoma, and 6.3 (3.3-9.9) years for rectal cancer; in the nonradiotherapy cohort, the median (IQR) time was 5.2 (3.1-8.4) years for bladder cancer, 5.7 (3.3-8.9) years for leukemia, 5.9 (3.3-9.0) years for lymphoma, and 5.0 (2.8-7.9) years for rectal cancer.

The number needed to harm, defined as the number of patients needed to receive radiotherapy for 1 additional patient to develop a second primary cancer, decreased with the amount of time from the prostate cancer diagnosis (Table 2). The number needed to harm was 333 patients for 0 to 5 years after diagnosis, 95 patients for 5 to 10 years after diagnosis, 53 patients for 10 to 15 years after diagnosis, and 40 patients for 15 to 20 years after diagnosis. In the multivariable analyses, patients in the radiotherapy cohort had a significantly higher risk of second primary cancer compared with patients in the nonradiotherapy cohort in the 5 years after diagnosis (hazard ratio [HR], 1.24; 95% CI, 1.13-1.37; *P* < .001). The risk was higher over the next 15 years (years 5-10: HR, 1.50 [95% CI, 1.36-1.65; *P* < .001]; years 10-15: HR, 1.59 [95% CI, 1.37-1.84; *P* < .001]; years 15-20: HR, 1.47 [95% CI, 1.08-2.01; *P* = .02]) (Table 3). Age at diagnosis (HR, 1.03 per additional year of age; 95% CI, 1.03-1.03 per additional year of age; *P* < .001) and higher Prostate Cancer Comorbidity Index score (score of 3-4 vs 0: HR, 1.12 [95% CI, 1.01-1.24; *P* = .04]; score of ≥5 vs 0: HR, 1.19 [95% CI, 1.11-1.28; *P* < .001]) were associated with a higher risk of developing a second primary cancer, whereas Black vs White race (HR, 0.76; 95% CI, 0.71-0.83; *P* < .001) and later vs earlier year of diagnosis (HR, 0.99 per additional year; 95% CI, 0.98-1.00 per additional year; *P* = .04) were associated with a lower risk of developing a second primary cancer.

**Table 2.** Second Primary Cancer–Free Survival and Number Needed to Harm After Prostate Cancer Diagnosis Among Radiotherapy and Nonradiotherapy Cohorts

Years after diagnosis	Patients, No.			Second primary cancer–free survival (95% CI)	Unadjusted NNH, No.^a^
	Beginning total	Second primary cancer	Censored		
Years 0-5					
Nonradiotherapy cohort	91 000	1036	13 291	0.9877 (0.9870-0.9884)	333
Radiotherapy cohort	52 886	757	6705	0.9847 (0.9836-0.9858)	
Years 5-10					
Nonradiotherapy cohort	76 673	831	37 379	0.9736 (0.9723-0.9747)	95
Radiotherapy cohort	45 424	771	20 706	0.9631 (0.9612-0.9649)	
Years 10-15					
Nonradiotherapy cohort	38 463	348	26 306	0.9602 (0.9583-0.9620)	53
Radiotherapy cohort	23 947	353	16 732	0.9413 (0.9383-0.9441)	
Years 15-20					
Nonradiotherapy cohort	11 809	87	11 722	0.9461 (0.9426-0.9495)	40
Radiotherapy cohort	6862	74	6788	0.9212 (0.9157-0.9263)	

Abbreviation: NNH, number needed to harm.

^a^ Number of patients needed to receive radiotherapy for 1 additional patient to develop a second primary cancer.

**Table 3.** Risk of Second Primary Cancer in Radiotherapy Cohort vs Nonradiotherapy Cohort^a^

Variable	Adjusted HR (95% CI)	*P* value
Radiotherapy vs nonradiotherapy		
Years 0-5	1.24 (1.13-1.37)	<.001
Years 5-10	1.50 (1.36-1.65)	<.001
Years 10-15	1.59 (1.37-1.84)	<.001
Years 15-20	1.47 (1.08-2.01)	.02
Risk per additional year of age at diagnosis	1.03 (1.03-1.03)	<.001
Race		
American Indian or Alaska Native	0.87 (0.56-1.35)	.53
Asian	0.96 (0.56-1.66)	.88
Black or African American	0.76 (0.71-0.83)	<.001
Native Hawaiian or Other Pacific Islander	0.70 (0.46-1.07)	.10
White	1 [Reference]	NA
Unknown	0.82 (0.71-0.94)	.003
Ethnicity		
Hispanic or Latino	0.89 (0.77-1.02)	.10
Not Hispanic or Latino	1 [Reference]	NA
Unknown	0.87 (0.72-1.06)	.16
D’Amico risk category^b^		
Low	1 [Reference]	NA
Intermediate	0.97 (0.90-1.04)	.40
High	1.02 (0.94-1.11)	.62
Undefined	0.92 (0.80-1.07)	.28
Agent Orange exposure	1.02 (0.93-1.11)	.71
Diagnosis year	0.99 (0.98-1.00)	.04
Median income based on patient zip code, $		
1-38 999	1 [Reference]	NA
39 000-47 999	1.04 (0.95-1.13)	.38
48 000-63 999	1.00 (0.91-1.10)	.97
≥64 000	1.05 (0.93-1.17)	.44
Missing	1.21 (0.55-2.66)	.64
Median educational level based on patient zip code		
High school or less	1 [Reference]	NA
Some college	0.95 (0.89-1.03)	.21
College or higher	1.04 (0.90-1.20)	.57
Missing	0.68 (.030-1.53)	.35
PCCI score^c^		
0	1 [Reference]	NA
1-2	1.03 (0.94-1.13)	.50
3-4	1.12 (1.01-1.24)	.04
≥5	1.19 (1.11-1.28)	<.001

Abbreviations: HR, hazard ratio; NA, not applicable; PCCI, Prostate Cancer Comorbidity Index.

^a^ Nonradiotherapy includes surgical procedures, active surveillance, medical management, or observation.

^b^ Likelihood of prostate cancer recurrence based on Gleason score, PSA value, and clinical tumor stage.

^c^ Higher scores indicate older age and greater number of comorbidities.

Sensitivity analyses limited to only those treated with primary radiotherapy or a surgical procedure revealed a consistent significantly higher risk in the first 5 years of a second primary cancer among patients who received radiotherapy compared with patients who received a surgical procedure (HR, 1.22; 95% CI, 1.07-1.39; *P* = .003), with a similar higher risk over time (years 5-10: HR, 1.54 [95% CI, 1.34-1.76; *P* < .001]; years 10-15: HR, 1.58 [95% CI, 1.31-1.92; *P* < .001]; years 15-20: HR, 1.44 [95% CI, 1.01-2.06; *P* = .046]) (eTable 1 in the Supplement). An additional sensitivity analysis including a smoking history covariate and limited to patients with available data yielded consistent results (years 0-5: HR, 1.22 [95% CI, 1.09-1.36; *P* < .001]; years 5-10: HR, 1.48 [95% CI, 1.32-1.67; *P* < .001]; years 10-15: HR, 1.58 [95% CI, 1.31-1.90; *P* < .001]; years 15-20: HR, 1.46 [95% CI, 0.93-2.29; *P* = .10]). (eTable 2 in the Supplement).

## Discussion

In this large national cohort study of men with localized prostate cancer who were followed up for a median of 9 years, treatment with primary radiotherapy was associated with a higher risk of developing a second primary cancer than treatment without radiotherapy. Although the higher incidence and risk of developing a second primary cancer were relatively small (occurring in only 3.0% of patients), the risk increased over time after completion of radiotherapy, and the number needed to harm notably decreased at 10 years after treatment. Moreover, prostate cancer is common, occurring in approximately 1 in 8 men in the US, and even a low-risk event can have consequences for a large absolute number of patients. Careful consideration of risks and benefits among accepted treatment modalities is therefore paramount. Our findings confirmed and updated conventional wisdom pertaining to a specific long-term risk of radiotherapy (especially ≥10 years after treatment) and supported consideration of the use of surgical procedures among otherwise healthy patients diagnosed in earlier decades of life compared with older patients or those with higher comorbid disease burden.

To our knowledge, this study of more than 100 000 patients with prostate cancer represents the largest cohort study to date assessing second primary cancers after prostate radiotherapy. The study cohort received treatment in the modern era of radiotherapy, whereas much of the previous literature, as reported in a systematic review,^16^ has focused on patients who received treatment from the 1970s to the early 2000s. During the 1970s to 1990s, patients were more likely to receive treatment with older radiotherapy techniques, such as 2-dimensional or 3-dimensional conformal radiotherapy, which are less conformal than techniques typically used today, such as intensity-modulated radiotherapy, volumetric arc radiotherapy, and stereotactic body radiotherapy. Although the CDW oncology data set lacks information regarding radiotherapy doses, fields, and techniques, our study period was one during which intensity-modulated radiotherapy, volumetric arc radiotherapy, and stereotactic body radiotherapy had been accepted practice standards for prostate radiotherapy.^17^ These techniques generally reduce the high radiation dose to normal tissues but may increase the low dose to other tissues, with some potential risk.^18^

The current study specifically compared a cohort of patients with prostate cancer who received primary radiotherapy with a cohort who did not receive radiotherapy, an important distinction from previous literature^16,19,20,21,22,23^ comparing patients with prostate cancer who received radiotherapy with the general population. Our findings in the modern radiotherapy era are supported by older data comparing patients with prostate cancer who received radiotherapy with those who did not, which also revealed an increased risk of bladder and rectal cancers after radiotherapy.^5,9,16,20,21^ Using the general population as a comparison group may be inappropriate because patients with prostate cancer are treated by urologists, who are more likely than primary care physicians to identify signs and symptoms of bladder cancer, for example. This phenomenon could play a role in the inconsistencies in the literature reporting variable incidence of bladder and rectal second primary cancers after prostate radiotherapy.^5,6,7,8,9,10,11^

In contrast to previous studies,^11,20,21^ bladder cancer was the most frequently diagnosed second primary cancer in the present study cohort, which may reflect the use of data from the VA population, among whom bladder cancer is the fourth most frequently diagnosed cancer.^24^ We also found that leukemias and lymphomas, which are not commonly addressed in the literature examining second primary cancers after prostate radiotherapy, were diagnosed more frequently than rectal cancer. Pelvic radiotherapy exposes the bone marrow to potentially high doses, and patients receiving prostate radiotherapy have been found to have an increased incidence of anemia.^25^ Radiation exposure is a risk factor for lymphoma,^26^ and a retrospective study^18^ of patients with prostate cancer who received treatment from 1999 to 2011 reported an increased risk of myelodysplastic syndrome and acute myeloid leukemia among patients receiving radiotherapy vs a surgical procedure (HR, 1.51). In that study,^18^ which used data from the Surveillance, Epidemiology, and End Results and Medicare databases, the median time to development of myelodysplastic syndrome or acute myeloid leukemia was approximately 3 years for both groups. Given the definition of radiotherapy-induced cancer, it is unclear whether these increased risks were associated with radiotherapy or whether the definition of radiotherapy-induced cancer needs to be updated. Our data contribute to the literature by revealing that primary radiotherapy for the treatment of prostate cancer was associated with a small but significantly higher risk of both leukemias and lymphomas compared with other treatment modalities. It is important for clinicians to be aware of this risk and follow up patients accordingly.

### Limitations

This study has limitations. Although we were able to classify patients as receiving vs not receiving radiotherapy, it is plausible that some misclassification occurred because patients who did not receive radiotherapy may have sought treatment outside the VA health care system. However, the VA Cancer Registrars capture initial prostate cancer treatments delivered outside of the VA health care system. In addition, some patients in the nonradiotherapy cohort may have received adjuvant or salvage radiotherapy in the years after their initial diagnosis, which may not have been captured. As with any retrospective cohort study, our data carry a risk of selection bias, and patients treated with radiotherapy may have had more comorbid disease with a higher risk of developing a second primary cancer. Although we were able to adjust for recorded comorbid disease counts using the well-established Prostate Cancer Comorbidity Index, it is possible that some comorbid diseases were not recorded in the patient records. We did not capture all potential risks associated with radiotherapy or surgical procedures, including outcomes that are difficult to ascertain using medical records but highly relevant to patients, such as sexual and urinary dysfunction. Although we performed a sensitivity analysis that included a smoking history covariate, information on pack-year smoking history was unreliable, and this variable was therefore not included as a confounder for a second primary bladder cancer.

## Conclusions

In this cohort study, patients with prostate cancer who received primary radiotherapy were more likely to develop a second primary cancer than those who did not receive radiotherapy, with increasing risk over time, especially 10 years or more after treatment. Overall, the incidence and risk of developing a second primary cancer were relatively low in both groups. Although the long-term toxic effects of radiotherapy are important to discuss when counseling patients on the risk-benefit profile of prostate radiotherapy, they need not deter physicians from recommending radiotherapy if appropriate. Patient selection and shared decision-making remain important when considering prostate cancer treatment options. Physicians caring for patients who have previously received radiotherapy for the treatment of prostate cancer might consider further evaluation if symptoms occur that potentially indicate the development of a second primary cancer.

## Notes

Supplement. **eTable 1.** Risk of Second Primary Cancer Among Patients Who Received Primary Radiotherapy vs a Surgical Procedure **eTable 2.** Smoking Sensitivity Analysis
