# Supplementary material for: Assessment of Second Primary Cancer Risk Among Men Receiving Primary Radiotherapy vs Surgery for the Treatment of Prostate Cancer
Source: JAMA Netw Open. 2022 Jul 28;5(7):e2223025. doi: 10.1001/jamanetworkopen.2022.23025 (PMC9335142; doi:10.1001/jamanetworkopen.2022.23025)
Supplement: Supplement. — eTable 1. Risk of Second Primary Cancer Among Patients Who Received Primary Radiotherapy vs a Surgical Procedure eTable 2. Smoking Sensitivity Analysis [file jamanetwopen-e2223025-s001.pdf]

Supplementary Online Content

Bagshaw HP, Arnow KD, Trickey AW, Leppert JT, Wren SM, Morris AM. Assessment of second primary cancer risk among men receiving primary radiotherapy vs surgery for the treatment of prostate cancer. *JAMA Netw Open*. 2022;5(7):e2223025. doi:10.1001/jamanetworkopen.2022.23025

eTable 1. Risk of Second Primary Cancer Among Patients Who Received Primary Radiotherapy vs a Surgical Procedure

eTable 2. Smoking Sensitivity Analysis

This supplementary material has been provided by the authors to give readers additional information about their work.

© 2022 Bagshaw HP et al. *JAMA Network Open*.

eTable 1. Risk of Second Primary Cancer Among Patients Who Received Primary Radiotherapy vs a Surgical Procedure (n = 84 104)

	Adjusted HR (95% CI)	p-value
Radiation (vs. surgery), years 0-5	1.22 (1.07, 1.39)	.003
Radiation (vs. surgery), years 5-10	1.54 (1.34, 1.76)	<.001
Radiation (vs. surgery), years 10-15	1.58 (1.31, 1.91)	<.001
Radiation (vs. surgery), years 15-20	1.44 (1.01, 2.06)	.046
Age at diagnosis, effect per additional year of age	1.03 (1.03, 1.04)	<.001
Race		
White	Referent	
American Indian or Alaska Native	0.90 (0.52, 1.55)	.67
Asian	0.86 (0.41, 1.80)	.68
Black or African American	0.76 (0.69, 0.84)	<.001
Native Hawaiian or Other Pacific Islander	0.81 (0.49, 1.32)	.40
Unknown	0.77 (0.65, 0.92)	.003
Ethnicity		
Not Hispanic or Latino	Referent	
Hispanic or Latino	0.78 (0.64, 0.95)	.012
Unknown	0.91 (0.72, 1.16)	.46
D'Amico Risk Category		
Low	Referent	
Intermediate	1.01 (0.93, 1.11)	.78
High	1.09 (0.98, 1.21)	.10
Undefined	0.9 (0.75, 1.07)	.23
Agent Orange exposure	1.05 (0.94, 1.16)	.42
Diagnosis year	0.99 (0.98, 1.00)	.009
Median income in patient zip		
1-38,999	Referent	
39,000-47,999	1.04 (0.94, 1.16)	.45
48,000-63,999	0.98 (0.88, 1.10)	.73
64,000+	1.02 (0.89, 1.18)	.73
Missing	0.9 (0.3, 2.7)	.85
Median education patient zip		
High School diploma or less	Referent	
Some college education	0.99 (0.90, 1.08)	.84
Undergraduate college degree or higher	1.09 (0.91, 1.30)	.36
Missing	0.87 (0.28, 2.70)	.82
PCCI		
0	Referent	
1-2	0.96 (0.85, 1.07)	.43
3-4	1.10 (0.97, 1.25)	.14
5+	1.14 (1.04, 1.24)	.003

© 2022 Bagshaw HP et al. *JAMA Network Open*.

[PCCI – prostate cancer-specific comorbidity index]

© 2022 Bagshaw HP et al. *JAMA Network Open*.

eTable 2. Smoking Sensitivity Analysis (n = 104 458)

	Adjusted HR (95% CI)	p-value
Radiation (vs. non-radiation), years 0-5	1.22 (1.09, 1.36)	<.001
Radiation (vs. non-radiation), years 5-10	1.48 (1.32, 1.67)	<.001
Radiation (vs. non-radiation), years 10-15	1.58 (1.31, 1.90)	<.001
Radiation (vs. non-radiation), years 15-20	1.46 (0.93, 2.29)	.10
Age at diagnosis, effect per additional year of age	1.03 (1.03, 1.04)	<.001
Race		
White	Referent	
American Indian or Alaska Native	0.61 (0.33, 1.14)	.12
Asian	1.16 (0.62, 2.17)	.63
Black or African American	0.76 (0.69, 0.83)	<.001
Native Hawaiian or Other Pacific Islander	0.75 (0.46, 1.23)	.25
Unknown	0.78 (0.66, 0.92)	.004
Ethnicity		
Not Hispanic or Latino	Referent	
Hispanic or Latino	1.04 (0.89, 1.22)	.61
Unknown	1.02 (0.81, 1.28)	.88
D'Amico Risk Category		
Low	Referent	
Intermediate	0.96 (0.88, 1.04)	.33
High	1.01 (0.91, 1.11)	.92
Undefined	0.9 (0.73, 1.10)	.30
Agent Orange exposure	0.98 (0.89, 1.09)	.75
Diagnosis year	0.98 (0.97, 1.00)	.004
Median income in patient zip		
1-38,999	Referent	
39,000-47,999	1.06 (0.95, 1.17)	.30
48,000-63,999	1.03 (0.92, 1.15)	.57
64,000+	1.07 (0.93, 1.23)	.34
Missing	1.52 (0.69, 3.38)	.30
Median education patient zip		
High School diploma or less	Referent	
Some college education	0.97 (0.89, 1.06)	.56
Undergraduate college degree or higher	0.99 (0.83, 1.18)	.87
Missing	0.53 (0.23, 1.21)	.13
PCCI		
0	Referent	
1-2	1.00 (0.90, 1.12)	.96
3-4	1.05 (0.92, 1.19)	.47
5+	1.10 (1.01, 1.20)	.023
Smoking history		

© 2022 Bagshaw HP et al. *JAMA Network Open*.

Current	Referent	
Former	0.76 (0.70, 0.84)	<.001
Never	0.60 (0.55, 0.66)	<.001

[PCCI – prostate cancer-specific comorbidity index]

© 2022 Bagshaw HP et al. *JAMA Network Open*.
